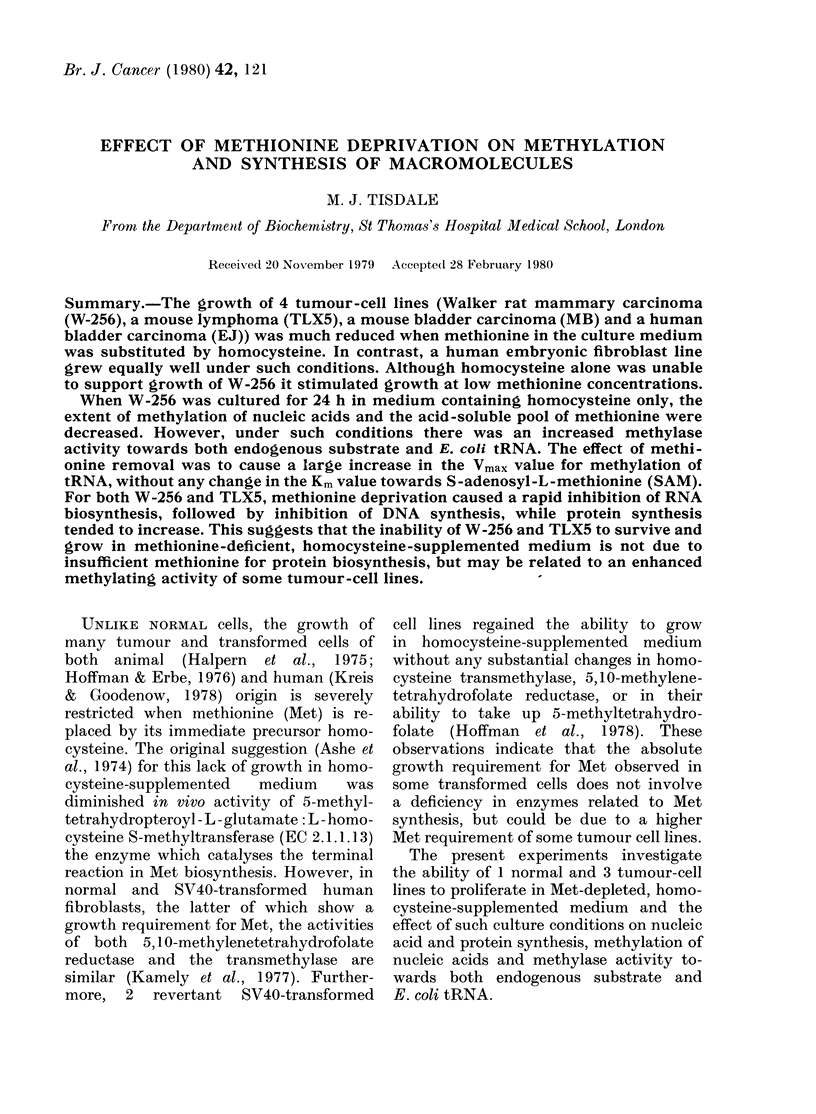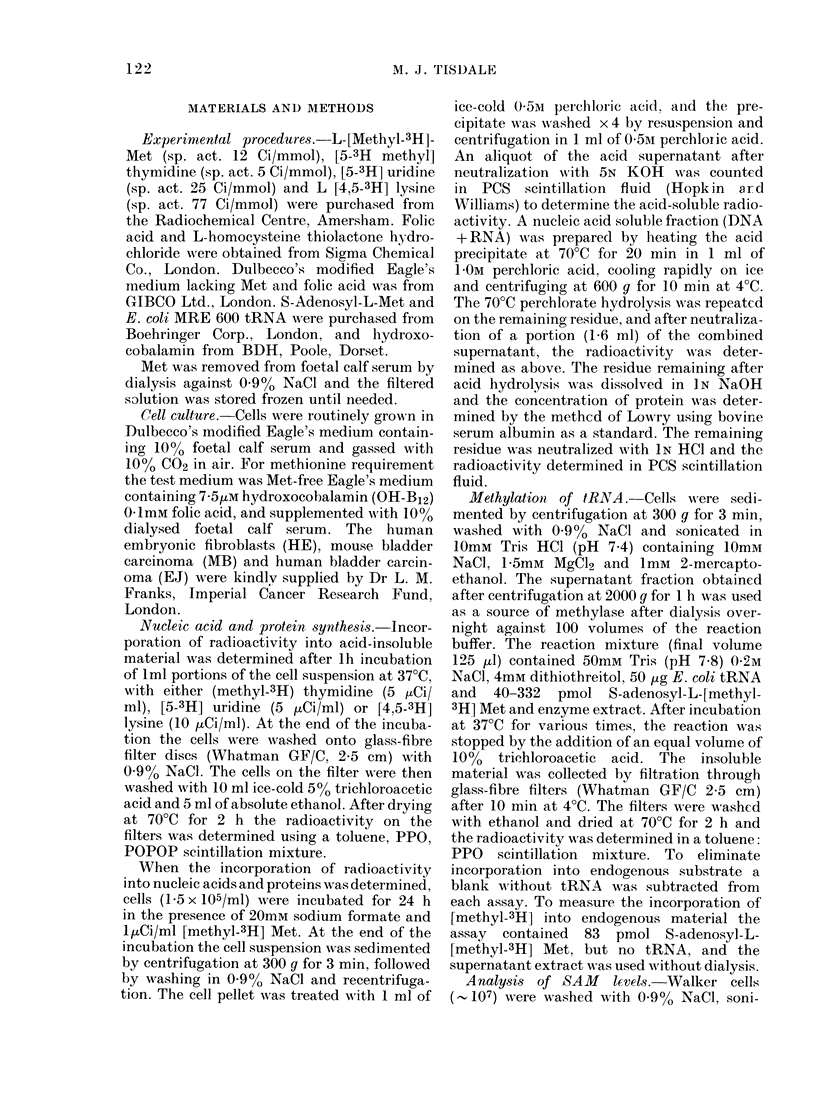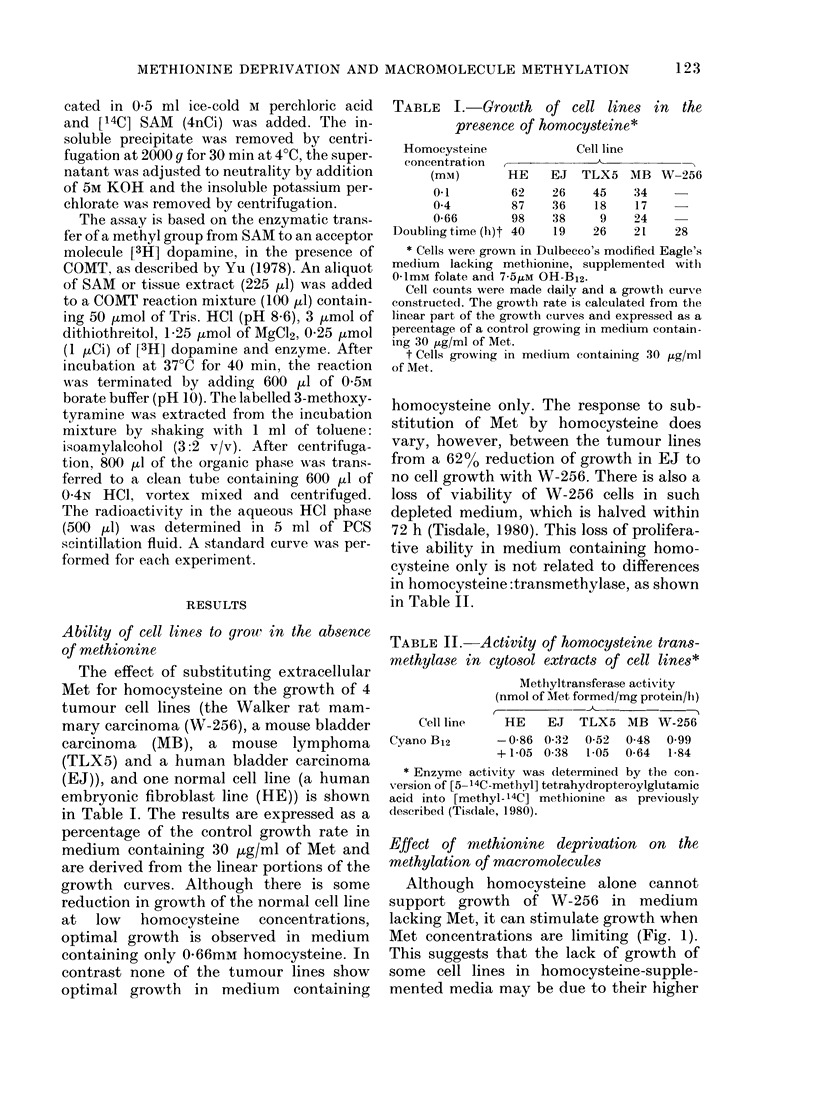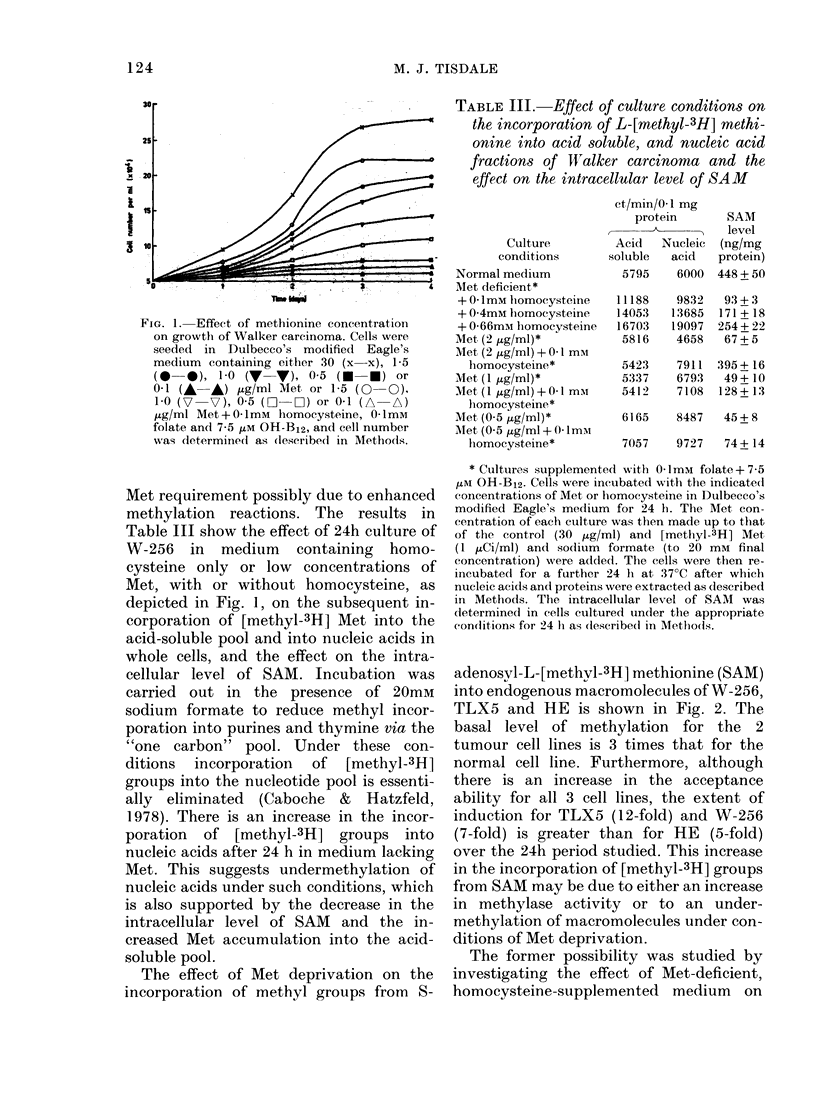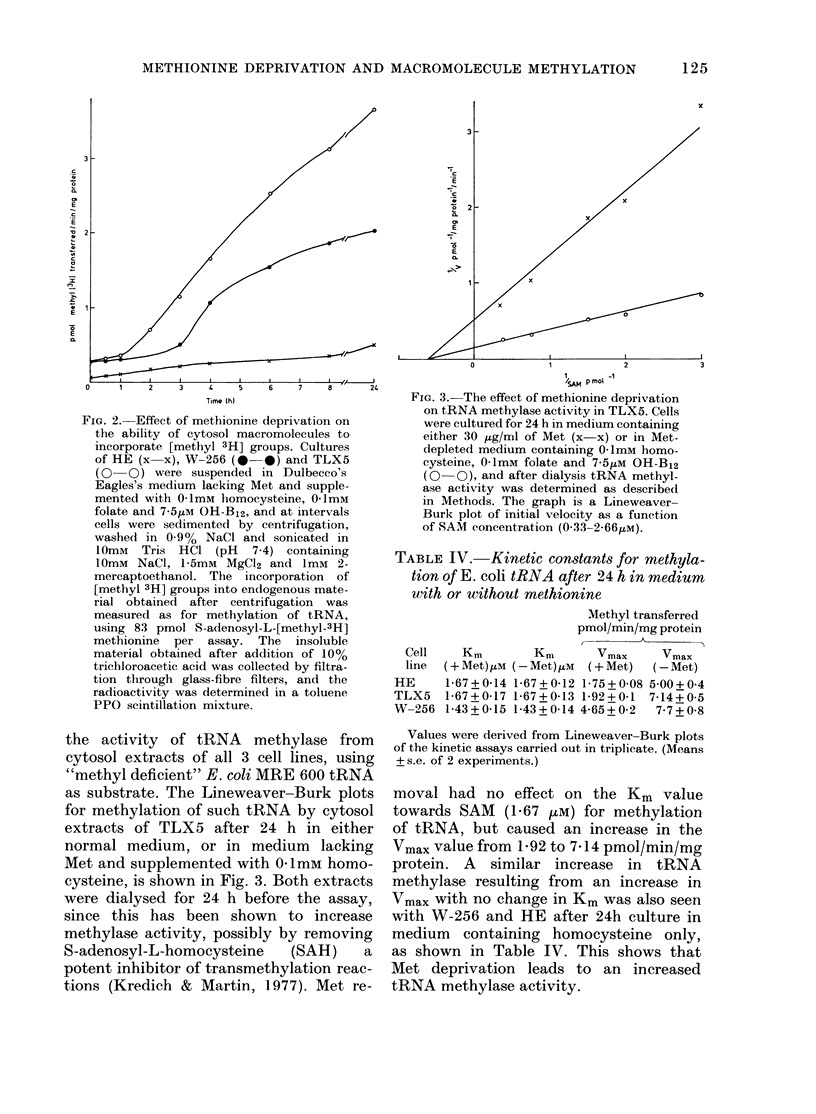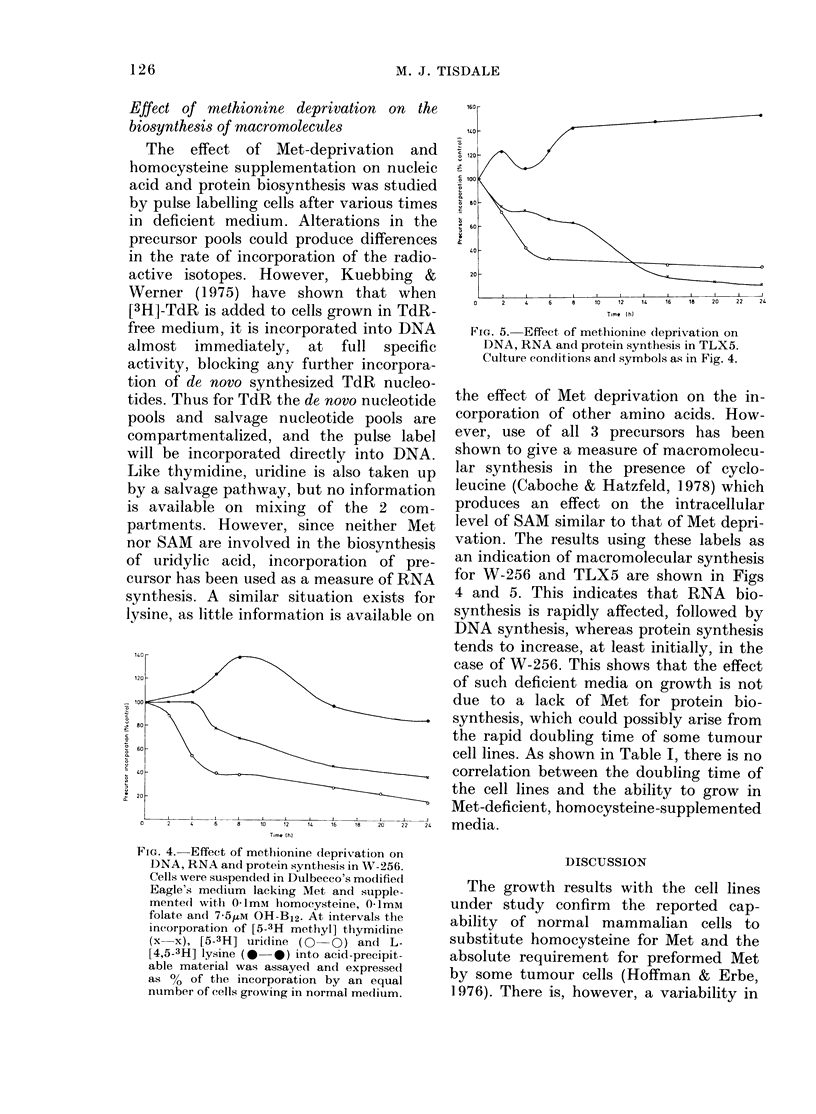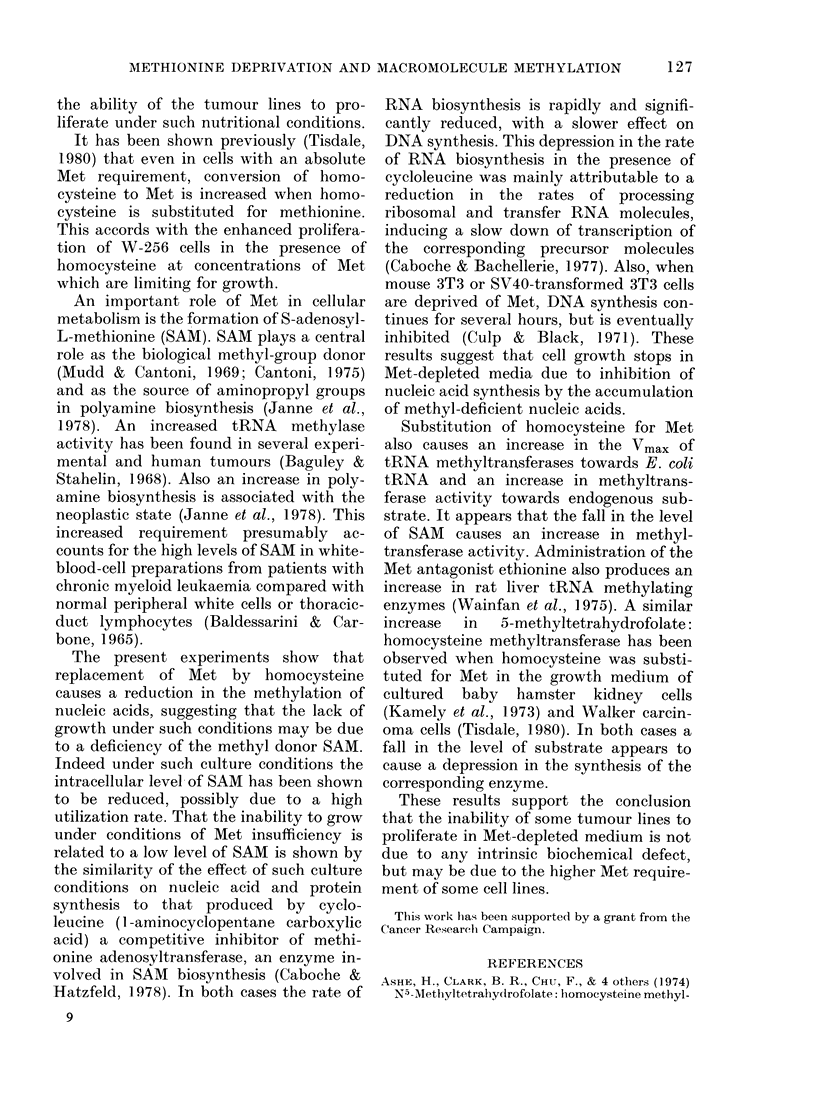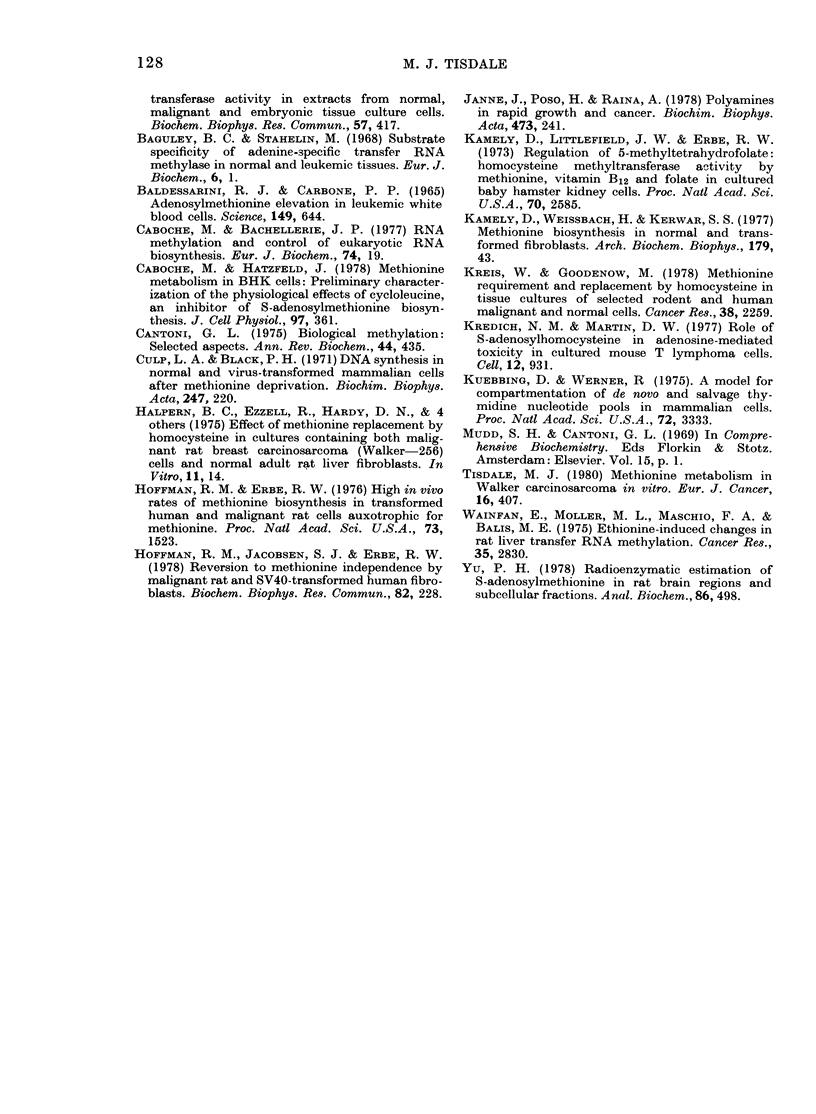# Effect of methionine deprivation on methylation and synthesis of macromolecules.

**DOI:** 10.1038/bjc.1980.210

**Published:** 1980-07

**Authors:** M. J. Tisdale

## Abstract

The growth of 4 tumour-cell lines (Walker rat mammary carcinoma (W-256), a mouse lymphoma (TLX5), a mouse bladder carcinoma (MB) and a human bladder carcinoma (EJ) was much reduced when methionine in the culture medium was substituted by homocysteine. In contrast, a human embryonic fibroblast line grew equally well under such conditions. Although homocysteine alone was unable to support growth of W-256 it stimulated growth at low methionine concentrations. When W-256 was cultured for 24 h in medium containing homocysteine only, the extent of methylation of nucleic acids and the acid-soluble pool of methionine were decreased. However, under such conditions there was an increased methylase activity towards both endogenous substrate and E. coli tRNA. The effect of methionine removal was to cause a large increase in the Vmax value for methylation of tRNA, without any change in the Km value towards S-adenosyl-L-methionine (SAM). For both W-256 and TLX5, methionine deprivation caused a rapid inhibition of RNA biosynthesis, followed by inhibition of DNA synthesis, while protein synthesis tended to increase. This suggests that the inability of W-256 and TLX5 to survive and grow in methionine-deficient, homocysteine-supplemented medium is not due to insufficient methionine for protein biosynthesis, but may be related to an enhanced methylating activity of some tumour-cell lines.


Br. J. Cancer (1980) 42, 121

### EFFECT OF METHIONINE DEPRIVATION ON METHYLATION AND SYNTHESIS OF MACROMOLECULES

M. J. TISDALE

*From the Department of Biochemistry, St Thomas's Hospital Medical School, London*

Received 20 November 1979 Accepted 28 February 1980

**Summary.—The growth of 4 tumour-cell lines (Walker rat mammary carcinoma (W-256), a mouse lymphoma (TLX5), a mouse bladder carcinoma (MB) and a human bladder carcinoma (EJ)) was much reduced when methionine in the culture medium was substituted by homocysteine. In contrast, a human embryonic fibroblast line grew equally well under such conditions. Although homocysteine alone was unable to support growth of W-256 it stimulated growth at low methionine concentrations.**

**When W-256 was cultured for 24 h in medium containing homocysteine only, the extent of methylation of nucleic acids and the acid-soluble pool of methionine were decreased. However, under such conditions there was an increased methylase activity towards both endogenous substrate and *E. coli* tRNA. The effect of methionine removal was to cause a large increase in the $V_{max}$ value for methylation of tRNA, without any change in the $K_m$ value towards S-adenosyl-L-methionine (SAM). For both W-256 and TLX5, methionine deprivation caused a rapid inhibition of RNA biosynthesis, followed by inhibition of DNA synthesis, while protein synthesis tended to increase. This suggests that the inability of W-256 and TLX5 to survive and grow in methionine-deficient, homocysteine-supplemented medium is not due to insufficient methionine for protein biosynthesis, but may be related to an enhanced methylating activity of some tumour-cell lines.**

UNLIKE NORMAL cells, the growth of many tumour and transformed cells of both animal (Halpern *et al.*, 1975; Hoffman & Erbe, 1976) and human (Kreis & Goodenow, 1978) origin is severely restricted when methionine (Met) is replaced by its immediate precursor homocysteine. The original suggestion (Ashe *et al.*, 1974) for this lack of growth in homocysteine-supplemented medium was diminished *in vivo* activity of 5-methyltetrahydropteroyl-L-glutamate:L-homocysteine S-methyltransferase (EC 2.1.1.13) the enzyme which catalyses the terminal reaction in Met biosynthesis. However, in normal and SV40-transformed human fibroblasts, the latter of which show a growth requirement for Met, the activities of both 5,10-methylenetetrahydrofolate reductase and the transmethylase are similar (Kamely *et al.*, 1977). Furthermore, 2 revertant SV40-transformed cell lines regained the ability to grow in homocysteine-supplemented medium without any substantial changes in homocysteine transmethylase, 5,10-methylenetetrahydrofolate reductase, or in their ability to take up 5-methyltetrahydrofolate (Hoffman *et al.*, 1978). These observations indicate that the absolute growth requirement for Met observed in some transformed cells does not involve a deficiency in enzymes related to Met synthesis, but could be due to a higher Met requirement of some tumour cell lines.

The present experiments investigate the ability of 1 normal and 3 tumour-cell lines to proliferate in Met-depleted, homocysteine-supplemented medium and the effect of such culture conditions on nucleic acid and protein synthesis, methylation of nucleic acids and methylase activity towards both endogenous substrate and *E. coli* tRNA.

### MATERIALS AND METHODS

*Experimental procedures.*—L-[Methyl-$^3$H]-Met (sp. act. 12 Ci/mmol), [5-$^3$H methyl] thymidine (sp. act. 5 Ci/mmol), [5-$^3$H] uridine (sp. act. 25 Ci/mmol) and L [4,5-$^3$H] lysine (sp. act. 77 Ci/mmol) were purchased from the Radiochemical Centre, Amersham. Folic acid and L-homocysteine thiolactone hydrochloride were obtained from Sigma Chemical Co., London. Dulbecco's modified Eagle's medium lacking Met and folic acid was from GIBCO Ltd., London. S-Adenosyl-L-Met and *E. coli* MRE 600 tRNA were purchased from Boehringer Corp., London, and hydroxocobalamin from BDH, Poole, Dorset.

Met was removed from foetal calf serum by dialysis against 0·9% NaCl and the filtered solution was stored frozen until needed.

*Cell culture.*—Cells were routinely grown in Dulbecco's modified Eagle's medium containing 10% foetal calf serum and gassed with 10% $CO_2$ in air. For methionine requirement the test medium was Met-free Eagle's medium containing 7·5$\mu$M hydroxocobalamin (OH-$B_{12}$) 0·1mM folic acid, and supplemented with 10% dialysed foetal calf serum. The human embryonic fibroblasts (HE), mouse bladder carcinoma (MB) and human bladder carcinoma (EJ) were kindly supplied by Dr L. M. Franks, Imperial Cancer Research Fund, London.

*Nucleic acid and protein synthesis.*—Incorporation of radioactivity into acid-insoluble material was determined after 1h incubation of 1ml portions of the cell suspension at 37°C, with either (methyl-$^3$H) thymidine (5 $\mu$Ci/ml), [5-$^3$H] uridine (5 $\mu$Ci/ml) or [4,5-$^3$H] lysine (10 $\mu$Ci/ml). At the end of the incubation the cells were washed onto glass-fibre filter discs (Whatman GF/C, 2·5 cm) with 0·9% NaCl. The cells on the filter were then washed with 10 ml ice-cold 5% trichloroacetic acid and 5 ml of absolute ethanol. After drying at 70°C for 2 h the radioactivity on the filters was determined using a toluene, PPO, POPOP scintillation mixture.

When the incorporation of radioactivity into nucleic acids and proteins was determined, cells (1·5 × $10^5$/ml) were incubated for 24 h in the presence of 20mM sodium formate and 1$\mu$Ci/ml [methyl-$^3$H] Met. At the end of the incubation the cell suspension was sedimented by centrifugation at 300 *g* for 3 min, followed by washing in 0·9% NaCl and recentrifugation. The cell pellet was treated with 1 ml of ice-cold 0·5M perchloric acid, and the precipitate was washed × 4 by resuspension and centrifugation in 1 ml of 0·5M perchloric acid. An aliquot of the acid supernatant after neutralization with 5N KOH was counted in PCS scintillation fluid (Hopkin and Williams) to determine the acid-soluble radioactivity. A nucleic acid soluble fraction (DNA + RNA) was prepared by heating the acid precipitate at 70°C for 20 min in 1 ml of 1·0M perchloric acid, cooling rapidly on ice and centrifuging at 600 *g* for 10 min at 4°C. The 70°C perchlorate hydrolysis was repeated on the remaining residue, and after neutralization of a portion (1·6 ml) of the combined supernatant, the radioactivity was determined as above. The residue remaining after acid hydrolysis was dissolved in 1N NaOH and the concentration of protein was determined by the method of Lowry using bovine serum albumin as a standard. The remaining residue was neutralized with 1N HCl and the radioactivity determined in PCS scintillation fluid.

*Methylation of tRNA.*—Cells were sedimented by centrifugation at 300 *g* for 3 min, washed with 0·9% NaCl and sonicated in 10mM Tris HCl (pH 7·4) containing 10mM NaCl, 1·5mM $MgCl_2$ and 1mM 2-mercaptoethanol. The supernatant fraction obtained after centrifugation at 2000 *g* for 1 h was used as a source of methylase after dialysis overnight against 100 volumes of the reaction buffer. The reaction mixture (final volume 125 $\mu$l) contained 50mM Tris (pH 7·8) 0·2M NaCl, 4mM dithiothreitol, 50 $\mu$g *E. coli* tRNA and 40–332 pmol S-adenosyl-L-[methyl-$^3$H] Met and enzyme extract. After incubation at 37°C for various times, the reaction was stopped by the addition of an equal volume of 10% trichloroacetic acid. The insoluble material was collected by filtration through glass-fibre filters (Whatman GF/C 2·5 cm) after 10 min at 4°C. The filters were washed with ethanol and dried at 70°C for 2 h and the radioactivity was determined in a toluene: PPO scintillation mixture. To eliminate incorporation into endogenous substrate a blank without tRNA was subtracted from each assay. To measure the incorporation of [methyl-$^3$H] into endogenous material the assay contained 83 pmol S-adenosyl-L-[methyl-$^3$H] Met, but no tRNA, and the supernatant extract was used without dialysis.

*Analysis of SAM levels.*—Walker cells (~$10^7$) were washed with 0·9% NaCl, sonicated in 0·5 ml ice-cold M perchloric acid and [$^{14}$C] SAM (4nCi) was added. The insoluble precipitate was removed by centrifugation at 2000 *g* for 30 min at 4°C, the supernatant was adjusted to neutrality by addition of 5M KOH and the insoluble potassium perchlorate was removed by centrifugation.

The assay is based on the enzymatic transfer of a methyl group from SAM to an acceptor molecule [$^3$H] dopamine, in the presence of COMT, as described by Yu (1978). An aliquot of SAM or tissue extract (225 $\mu$l) was added to a COMT reaction mixture (100 $\mu$l) containing 50 $\mu$mol of Tris. HCl (pH 8·6), 3 $\mu$mol of dithiothreitol, 1·25 $\mu$mol of $MgCl_2$, 0·25 $\mu$mol (1 $\mu$Ci) of [$^3$H] dopamine and enzyme. After incubation at 37°C for 40 min, the reaction was terminated by adding 600 $\mu$l of 0·5M borate buffer (pH 10). The labelled 3-methoxytyramine was extracted from the incubation mixture by shaking with 1 ml of toluene: isoamylalcohol (3:2 v/v). After centrifugation, 800 $\mu$l of the organic phase was transferred to a clean tube containing 600 $\mu$l of 0·4N HCl, vortex mixed and centrifuged. The radioactivity in the aqueous HCl phase (500 $\mu$l) was determined in 5 ml of PCS scintillation fluid. A standard curve was performed for each experiment.

### RESULTS

### *Ability of cell lines to grow in the absence of methionine*

The effect of substituting extracellular Met for homocysteine on the growth of 4 tumour cell lines (the Walker rat mammary carcinoma (W-256), a mouse bladder carcinoma (MB), a mouse lymphoma (TLX5) and a human bladder carcinoma (EJ)), and one normal cell line (a human embryonic fibroblast line (HE)) is shown in Table I. The results are expressed as a percentage of the control growth rate in medium containing 30 $\mu$g/ml of Met and are derived from the linear portions of the growth curves. Although there is some reduction in growth of the normal cell line at low homocysteine concentrations, optimal growth is observed in medium containing only 0·66mM homocysteine. In contrast none of the tumour lines show optimal growth in medium containing homocysteine only. The response to substitution of Met by homocysteine does vary, however, between the tumour lines from a 62% reduction of growth in EJ to no cell growth with W-256. There is also a loss of viability of W-256 cells in such depleted medium, which is halved within 72 h (Tisdale, 1980). This loss of proliferative ability in medium containing homocysteine only is not related to differences in homocysteine:transmethylase, as shown in Table II.

TABLE I.—*Growth of cell lines in the presence of homocysteine**

| Homocysteine concentration (mM) | Cell line HE | EJ | TLX5 | MB | W-256 |
|---|---|---|---|---|---|
| 0·1 | 62 | 26 | 45 | 34 | — |
| 0·4 | 87 | 36 | 18 | 17 | — |
| 0·66 | 98 | 38 | 9 | 24 | — |
| Doubling time (h)† | 40 | 19 | 26 | 21 | 28 |

* Cells were grown in Dulbecco's modified Eagle's medium lacking methionine, supplemented with 0·1mM folate and 7·5$\mu$M OH-$B_{12}$.

Cell counts were made daily and a growth curve constructed. The growth rate is calculated from the linear part of the growth curves and expressed as a percentage of a control growing in medium containing 30 $\mu$g/ml of Met.

† Cells growing in medium containing 30 $\mu$g/ml of Met.

TABLE II.—*Activity of homocysteine transmethylase in cytosol extracts of cell lines**

| Cell line | Methyltransferase activity (nmol of Met formed/mg protein/h) HE | EJ | TLX5 | MB | W-256 |
|---|---|---|---|---|---|
| Cyano $B_{12}$ − | 0·86 | 0·32 | 0·52 | 0·48 | 0·99 |
| + | 1·05 | 0·38 | 1·05 | 0·64 | 1·84 |

* Enzyme activity was determined by the conversion of [5-$^{14}$C-methyl] tetrahydropteroylglutamic acid into [methyl-$^{14}$C] methionine as previously described (Tisdale, 1980).

### *Effect of methionine deprivation on the methylation of macromolecules*

Although homocysteine alone cannot support growth of W-256 in medium lacking Met, it can stimulate growth when Met concentrations are limiting (Fig. 1). This suggests that the lack of growth of some cell lines in homocysteine-supplemented media may be due to their higher

FIG. 1.—Effect of methionine concentration on growth of Walker carcinoma. Cells were seeded in Dulbecco's modified Eagle's medium containing either 30 (x—x), 1·5 (●—●), 1·0 (▼—▼), 0·5 (■—■) or 0·1 (▲—▲) μg/ml Met or 1·5 (○—○), 1·0 (▽—▽), 0·5 (□—□) or 0·1 (△—△) μg/ml Met+0·1mM homocysteine, 0·1mM folate and 7·5 μM OH-$B_{12}$, and cell number was determined as described in Methods.

TABLE III.—*Effect of culture conditions on the incorporation of L-[methyl-$^3$H] methionine into acid soluble, and nucleic acid fractions of Walker carcinoma and the effect on the intracellular level of SAM*

| Culture conditions | ct/min/0·1 mg protein: Acid soluble | Nucleic acid | SAM level (ng/mg protein) |
|---|---|---|---|
| Normal medium | 5795 | 6000 | 448 ± 50 |
| Met deficient* | | | |
| + 0·1mM homocysteine | 11188 | 9832 | 93 ± 3 |
| + 0·4mM homocysteine | 14053 | 13685 | 171 ± 18 |
| + 0·66mM homocysteine | 16703 | 19097 | 254 ± 22 |
| Met (2 μg/ml)* | 5816 | 4658 | 67 ± 5 |
| Met (2 μg/ml) + 0·1 mM homocysteine* | 5423 | 7911 | 395 ± 16 |
| Met (1 μg/ml)* | 5337 | 6793 | 49 ± 10 |
| Met (1 μg/ml) + 0·1 mM homocysteine* | 5412 | 7108 | 128 ± 13 |
| Met (0·5 μg/ml)* | 6165 | 8487 | 45 ± 8 |
| Met (0·5 μg/ml + 0·1mM homocysteine* | 7057 | 9727 | 74 ± 14 |

* Cultures supplemented with 0·1mM folate + 7·5 μM OH-$B_{12}$. Cells were incubated with the indicated concentrations of Met or homocysteine in Dulbecco's modified Eagle's medium for 24 h. The Met concentration of each culture was then made up to that of the control (30 μg/ml) and [methyl-$^3$H] Met (1 μCi/ml) and sodium formate (to 20 mM final concentration) were added. The cells were then reincubated for a further 24 h at 37°C after which nucleic acids and proteins were extracted as described in Methods. The intracellular level of SAM was determined in cells cultured under the appropriate conditions for 24 h as described in Methods.

Met requirement possibly due to enhanced methylation reactions. The results in Table III show the effect of 24h culture of W-256 in medium containing homocysteine only or low concentrations of Met, with or without homocysteine, as depicted in Fig. 1, on the subsequent incorporation of [methyl-$^3$H] Met into the acid-soluble pool and into nucleic acids in whole cells, and the effect on the intracellular level of SAM. Incubation was carried out in the presence of 20mM sodium formate to reduce methyl incorporation into purines and thymine *via* the "one carbon" pool. Under these conditions incorporation of [methyl-$^3$H] groups into the nucleotide pool is essentially eliminated (Caboche & Hatzfeld, 1978). There is an increase in the incorporation of [methyl-$^3$H] groups into nucleic acids after 24 h in medium lacking Met. This suggests undermethylation of nucleic acids under such conditions, which is also supported by the decrease in the intracellular level of SAM and the increased Met accumulation into the acid-soluble pool.

The effect of Met deprivation on the incorporation of methyl groups from S-adenosyl-L-[methyl-$^3$H] methionine (SAM) into endogenous macromolecules of W-256, TLX5 and HE is shown in Fig. 2. The basal level of methylation for the 2 tumour cell lines is 3 times that for the normal cell line. Furthermore, although there is an increase in the acceptance ability for all 3 cell lines, the extent of induction for TLX5 (12-fold) and W-256 (7-fold) is greater than for HE (5-fold) over the 24h period studied. This increase in the incorporation of [methyl-$^3$H] groups from SAM may be due to either an increase in methylase activity or to an undermethylation of macromolecules under conditions of Met deprivation.

The former possibility was studied by investigating the effect of Met-deficient, homocysteine-supplemented medium on

FIG. 2.—Effect of methionine deprivation on the ability of cytosol macromolecules to incorporate [methyl $^{3}H$] groups. Cultures of HE (x—x), W-256 (●—●) and TLX5 (○—○) were suspended in Dulbecco's Eagles's medium lacking Met and supplemented with 0·1mM homocysteine, 0·1mM folate and 7·5μM OH-$B_{12}$, and at intervals cells were sedimented by centrifugation, washed in 0·9% NaCl and sonicated in 10mM Tris HCl (pH 7·4) containing 10mM NaCl, 1·5mM $MgCl_2$ and 1mM 2-mercaptoethanol. The incorporation of [methyl $^{3}H$] groups into endogenous material obtained after centrifugation was measured as for methylation of tRNA, using 83 pmol S-adenosyl-L-[methyl-$^{3}H$] methionine per assay. The insoluble material obtained after addition of 10% trichloroacetic acid was collected by filtration through glass-fibre filters, and the radioactivity was determined in a toluene PPO scintillation mixture.

FIG. 3.—The effect of methionine deprivation on tRNA methylase activity in TLX5. Cells were cultured for 24 h in medium containing either 30 μg/ml of Met (x—x) or in Met-depleted medium containing 0·1mM homocysteine, 0·1mM folate and 7·5μM OH-$B_{12}$ (○—○), and after dialysis tRNA methylase activity was determined as described in Methods. The graph is a Lineweaver–Burk plot of initial velocity as a function of SAM concentration (0·33–2·66μM).

the activity of tRNA methylase from cytosol extracts of all 3 cell lines, using "methyl deficient" *E. coli* MRE 600 tRNA as substrate. The Lineweaver–Burk plots for methylation of such tRNA by cytosol extracts of TLX5 after 24 h in either normal medium, or in medium lacking Met and supplemented with 0·1mM homocysteine, is shown in Fig. 3. Both extracts were dialysed for 24 h before the assay, since this has been shown to increase methylase activity, possibly by removing S-adenosyl-L-homocysteine (SAH) a potent inhibitor of transmethylation reactions (Kredich & Martin, 1977). Met removal had no effect on the $K_m$ value towards SAM (1·67 μM) for methylation of tRNA, but caused an increase in the $V_{max}$ value from 1·92 to 7·14 pmol/min/mg protein. A similar increase in tRNA methylase resulting from an increase in $V_{max}$ with no change in $K_m$ was also seen with W-256 and HE after 24h culture in medium containing homocysteine only, as shown in Table IV. This shows that Met deprivation leads to an increased tRNA methylase activity.

TABLE IV.—*Kinetic constants for methylation of* E. coli *tRNA after 24 h in medium with or without methionine*

| Cell line | $K_m$ (+Met)μM | $K_m$ (−Met)μM | Methyl transferred pmol/min/mg protein $V_{max}$ (+Met) | $V_{max}$ (−Met) |
|---|---|---|---|---|
| HE | 1·67±0·14 | 1·67±0·12 | 1·75±0·08 | 5·00±0·4 |
| TLX5 | 1·67±0·17 | 1·67±0·13 | 1·92±0·1 | 7·14±0·5 |
| W-256 | 1·43±0·15 | 1·43±0·14 | 4·65±0·2 | 7·7±0·8 |

Values were derived from Lineweaver–Burk plots of the kinetic assays carried out in triplicate. (Means ±s.e. of 2 experiments.)

*Effect of methionine deprivation on the biosynthesis of macromolecules*

The effect of Met-deprivation and homocysteine supplementation on nucleic acid and protein biosynthesis was studied by pulse labelling cells after various times in deficient medium. Alterations in the precursor pools could produce differences in the rate of incorporation of the radioactive isotopes. However, Kuebbing & Werner (1975) have shown that when [$^3$H]-TdR is added to cells grown in TdR-free medium, it is incorporated into DNA almost immediately, at full specific activity, blocking any further incorporation of *de novo* synthesized TdR nucleotides. Thus for TdR the *de novo* nucleotide pools and salvage nucleotide pools are compartmentalized, and the pulse label will be incorporated directly into DNA. Like thymidine, uridine is also taken up by a salvage pathway, but no information is available on mixing of the 2 compartments. However, since neither Met nor SAM are involved in the biosynthesis of uridylic acid, incorporation of precursor has been used as a measure of RNA synthesis. A similar situation exists for lysine, as little information is available on the effect of Met deprivation on the incorporation of other amino acids. However, use of all 3 precursors has been shown to give a measure of macromolecular synthesis in the presence of cycloleucine (Caboche & Hatzfeld, 1978) which produces an effect on the intracellular level of SAM similar to that of Met deprivation. The results using these labels as an indication of macromolecular synthesis for W-256 and TLX5 are shown in Figs 4 and 5. This indicates that RNA biosynthesis is rapidly affected, followed by DNA synthesis, whereas protein synthesis tends to increase, at least initially, in the case of W-256. This shows that the effect of such deficient media on growth is not due to a lack of Met for protein biosynthesis, which could possibly arise from the rapid doubling time of some tumour cell lines. As shown in Table I, there is no correlation between the doubling time of the cell lines and the ability to grow in Met-deficient, homocysteine-supplemented media.

FIG. 4.—Effect of methionine deprivation on DNA, RNA and protein synthesis in W-256. Cells were suspended in Dulbecco's modified Eagle's medium lacking Met and supplemented with 0·1mM homocysteine, 0·1mM folate and 7·5$\mu$M OH-$B_{12}$. At intervals the incorporation of [5-$^3$H methyl] thymidine (x—x), [5-$^3$H] uridine (○—○) and L-[4,5-$^3$H] lysine (●—●) into acid-precipitable material was assayed and expressed as % of the incorporation by an equal number of cells growing in normal medium.

FIG. 5.—Effect of methionine deprivation on DNA, RNA and protein synthesis in TLX5. Culture conditions and symbols as in Fig. 4.

### DISCUSSION

The growth results with the cell lines under study confirm the reported capability of normal mammalian cells to substitute homocysteine for Met and the absolute requirement for preformed Met by some tumour cells (Hoffman & Erbe, 1976). There is, however, a variability in the ability of the tumour lines to proliferate under such nutritional conditions.

It has been shown previously (Tisdale, 1980) that even in cells with an absolute Met requirement, conversion of homocysteine to Met is increased when homocysteine is substituted for methionine. This accords with the enhanced proliferation of W-256 cells in the presence of homocysteine at concentrations of Met which are limiting for growth.

An important role of Met in cellular metabolism is the formation of S-adenosyl-L-methionine (SAM). SAM plays a central role as the biological methyl-group donor (Mudd & Cantoni, 1969; Cantoni, 1975) and as the source of aminopropyl groups in polyamine biosynthesis (Janne *et al.*, 1978). An increased tRNA methylase activity has been found in several experimental and human tumours (Baguley & Stahelin, 1968). Also an increase in polyamine biosynthesis is associated with the neoplastic state (Janne *et al.*, 1978). This increased requirement presumably accounts for the high levels of SAM in white-blood-cell preparations from patients with chronic myeloid leukaemia compared with normal peripheral white cells or thoracic-duct lymphocytes (Baldessarini & Carbone, 1965).

The present experiments show that replacement of Met by homocysteine causes a reduction in the methylation of nucleic acids, suggesting that the lack of growth under such conditions may be due to a deficiency of the methyl donor SAM. Indeed under such culture conditions the intracellular level of SAM has been shown to be reduced, possibly due to a high utilization rate. That the inability to grow under conditions of Met insufficiency is related to a low level of SAM is shown by the similarity of the effect of such culture conditions on nucleic acid and protein synthesis to that produced by cycloleucine (1-aminocyclopentane carboxylic acid) a competitive inhibitor of methionine adenosyltransferase, an enzyme involved in SAM biosynthesis (Caboche & Hatzfeld, 1978). In both cases the rate of RNA biosynthesis is rapidly and significantly reduced, with a slower effect on DNA synthesis. This depression in the rate of RNA biosynthesis in the presence of cycloleucine was mainly attributable to a reduction in the rates of processing ribosomal and transfer RNA molecules, inducing a slow down of transcription of the corresponding precursor molecules (Caboche & Bachellerie, 1977). Also, when mouse 3T3 or SV40-transformed 3T3 cells are deprived of Met, DNA synthesis continues for several hours, but is eventually inhibited (Culp & Black, 1971). These results suggest that cell growth stops in Met-depleted media due to inhibition of nucleic acid synthesis by the accumulation of methyl-deficient nucleic acids.

Substitution of homocysteine for Met also causes an increase in the $V_{max}$ of tRNA methyltransferases towards *E. coli* tRNA and an increase in methyltransferase activity towards endogenous substrate. It appears that the fall in the level of SAM causes an increase in methyltransferase activity. Administration of the Met antagonist ethionine also produces an increase in rat liver tRNA methylating enzymes (Wainfan *et al.*, 1975). A similar increase in 5-methyltetrahydrofolate: homocysteine methyltransferase has been observed when homocysteine was substituted for Met in the growth medium of cultured baby hamster kidney cells (Kamely *et al.*, 1973) and Walker carcinoma cells (Tisdale, 1980). In both cases a fall in the level of substrate appears to cause a depression in the synthesis of the corresponding enzyme.

These results support the conclusion that the inability of some tumour lines to proliferate in Met-depleted medium is not due to any intrinsic biochemical defect, but may be due to the higher Met requirement of some cell lines.

This work has been supported by a grant from the Cancer Research Campaign.

### REFERENCES

ASHE, H., CLARK, B. R., CHU, F., & 4 others (1974) $N^5$-Methyltetrahydrofolate: homocysteine methyltransferase activity in extracts from normal, malignant and embryonic tissue culture cells. *Biochem. Biophys. Res. Commun.*, **57**, 417.

BAGULEY, B. C. & STAHELIN, M. (1968) Substrate specificity of adenine-specific transfer RNA methylase in normal and leukemic tissues. *Eur. J. Biochem.*, **6**, 1.

BALDESSARINI, R. J. & CARBONE, P. P. (1965) Adenosylmethionine elevation in leukemic white blood cells. *Science*, **149**, 644.

CABOCHE, M. & BACHELLERIE, J. P. (1977) RNA methylation and control of eukaryotic RNA biosynthesis. *Eur. J. Biochem.*, **74**, 19.

CABOCHE, M. & HATZFELD, J. (1978) Methionine metabolism in BHK cells: Preliminary characterization of the physiological effects of cycloleucine, an inhibitor of S-adenosylmethionine biosynthesis. *J. Cell Physiol.*, **97**, 361.

CANTONI, G. L. (1975) Biological methylation: Selected aspects. *Ann. Rev. Biochem.*, **44**, 435.

CULP, L. A. & BLACK, P. H. (1971) DNA synthesis in normal and virus-transformed mammalian cells after methionine deprivation. *Biochim. Biophys. Acta*, **247**, 220.

HALPERN, B. C., EZZELL, R., HARDY, D. N., & 4 others (1975) Effect of methionine replacement by homocysteine in cultures containing both malignant rat breast carcinosarcoma (Walker—256) cells and normal adult rat liver fibroblasts. *In Vitro*, **11**, 14.

HOFFMAN, R. M. & ERBE, R. W. (1976) High *in vivo* rates of methionine biosynthesis in transformed human and malignant rat cells auxotrophic for methionine. *Proc. Natl Acad. Sci. U.S.A.*, **73**, 1523.

HOFFMAN, R. M., JACOBSEN, S. J. & ERBE, R. W. (1978) Reversion to methionine independence by malignant rat and SV40-transformed human fibroblasts. *Biochem. Biophys. Res. Commun.*, **82**, 228.

JANNE, J., POSO, H. & RAINA, A. (1978) Polyamines in rapid growth and cancer. *Biochim. Biophys. Acta*, **473**, 241.

KAMELY, D., LITTLEFIELD, J. W. & ERBE, R. W. (1973) Regulation of 5-methyltetrahydrofolate: homocysteine methyltransferase activity by methionine, vitamin $B_{12}$ and folate in cultured baby hamster kidney cells. *Proc. Natl Acad. Sci. U.S.A.*, **70**, 2585.

KAMELY, D., WEISSBACH, H. & KERWAR, S. S. (1977) Methionine biosynthesis in normal and transformed fibroblasts. *Arch. Biochem. Biophys.*, **179**, 43.

KREIS, W. & GOODENOW, M. (1978) Methionine requirement and replacement by homocysteine in tissue cultures of selected rodent and human malignant and normal cells. *Cancer Res.*, **38**, 2259.

KREDICH, N. M. & MARTIN, D. W. (1977) Role of S-adenosylhomocysteine in adenosine-mediated toxicity in cultured mouse T lymphoma cells. *Cell*, **12**, 931.

KUEBBING, D. & WERNER, R (1975). A model for compartmentation of *de novo* and salvage thymidine nucleotide pools in mammalian cells. *Proc. Natl Acad. Sci. U.S.A.*, **72**, 3333.

MUDD, S. H. & CANTONI, G. L. (1969) In *Comprehensive Biochemistry*. Eds Florkin & Stotz. Amsterdam: Elsevier. Vol. 15, p. 1.

TISDALE, M. J. (1980) Methionine metabolism in Walker carcinosarcoma *in vitro*. *Eur. J. Cancer*, **16**, 407.

WAINFAN, E., MOLLER, M. L., MASCHIO, F. A. & BALIS, M. E. (1975) Ethionine-induced changes in rat liver transfer RNA methylation. *Cancer Res.*, **35**, 2830.

YU, P. H. (1978) Radioenzymatic estimation of S-adenosylmethionine in rat brain regions and subcellular fractions. *Anal. Biochem.*, **86**, 498.